# Smoking-associated *AHRR* demethylation in cord blood DNA: impact of CD235a+ nucleated red blood cells

**DOI:** 10.1186/s13148-019-0686-1

**Published:** 2019-06-10

**Authors:** Matthew A. Bergens, Gary S. Pittman, Isabel J. B. Thompson, Michelle R. Campbell, Xuting Wang, Cathrine Hoyo, Douglas A. Bell

**Affiliations:** 1 Environmental Epigenomics and Disease Group, Immunity, Inflammation, and Disease Laboratory, National Institute of Environmental Health Sciences, National Institutes of Health, Research Triangle Park, NC 27709 USA; 2 Epidemiology and Environmental Epigenomics Laboratory, North Carolina State University, Raleigh, NC 27695 USA

## Abstract

**Background:**

Numerous studies have demonstrated that DNA methylation levels in the aryl hydrocarbon receptor repressor (*AHRR*) gene measured in cord blood are significantly associated with prenatal tobacco smoke exposure and can be used as a fetal exposure biomarker. The mechanism driving this demethylation has not been determined and it is unclear if all cord blood cell types are impacted. Nucleated red blood cells (nRBCs/CD235a+ cells) are developmentally immature RBCs that display genome-wide hypomethylation and are observed at increased frequency in the cord blood of smoking mothers. We tested if *AHRR* methylation levels in CD235a+ nRBCs or nRBC counts influenced *AHRR* methylation in whole cord blood.

**Methods:**

Cord blood was collected from smoking (*n* = 34) and nonsmoking (*n* = 19) mothers and DNA was prepared from whole cord blood, isolated CD235a+ nRBCs, and CD14+ monocytes. *AHRR* methylation in cord blood DNA was measured using Illumina 850K arrays (cg05575921, chr5:373378). Pyrosequencing was used to compare methylation levels among cord blood, CD235a+, and CD14+ cells. We measured nRBC percentages using conventional complete blood counts and estimated percent nRBCs by a deconvolution model.

**Results:**

Methylation levels in *AHRR* were significantly lower in nRBCs relative to whole cord blood and CD14+ monocytes. While *AHRR* methylation levels in the cell types were significantly correlated across all subjects, methylation values at the chr5:373378 CpG averaged 14.6% lower in nRBCs (range 0.4 to 24.8%; *p* = 3.8E−13) relative to CD14+, with nonsmokers showing a significantly greater hypomethylation (− 4.1%, *p* = 1.8E−02). Methylation level at the *AHRR* chr5:373378 CpG was strongly associated with self-reported smoking in both CD14+ monocytes (*t* test *p* = 5.7E−09) and nRBCs (*p* = 4.8E−08), as well as cotinine levels (regression *p* = 1.1E−07 and *p* = 3.6E−04, respectively). For subjects with whole blood 850K data, robust linear regression models adjusting for estimated cell type composition, either including nRBCs counts or estimates, modestly increased the association between smoking and cg05575921 methylation.

**Conclusions:**

Prenatal smoke exposure was highly significantly associated with *AHRR* methylation in cord blood, CD14+ monocytes, and CD235a+ nRBCs. *AHRR* methylation levels in nRBCs and nRBC counts had minimal effect on cord blood methylation measurements. However, regression models using estimated nRBCs or actual nRBC counts outperformed those lacking these covariates.

**Electronic supplementary material:**

The online version of this article (10.1186/s13148-019-0686-1) contains supplementary material, which is available to authorized users.

## Introduction

Tobacco use during pregnancy is a major risk factor for adverse outcomes in children [1], and cytosine DNA methylation levels in cord blood have emerged as useful biomarkers of prenatal tobacco smoke exposure [2]. While smoking is strongly associated with epigenetic modifications in the DNA isolated from cord blood [3–5], the relative levels of epigenetic modification in individual immune cell types within cord blood is unexplored. In adults, myeloid cell types (granulocytes and monocytes) display greater sensitivity to tobacco smoke-associated DNA methylation alterations relative to lymphoid lineage cells [6, 7]; however, it is unclear if any cord blood cell types show similar sensitivity. The frequent presence of immature erythroid-lineage, nucleated red blood cells (nRBCs) in cord blood and their potential for modulating DNA methylation measurements in whole cord blood [4, 8] is of interest because nRBCs contain genomic DNA that is rapidly undergoing genome-wide demethylation and enucleation during erythropoiesis [9–12]. Thus, it has been suggested that methylation levels of genomic DNA present in nRBCs could affect or confound observations of methylation changes in whole cord blood DNA or other cell types [8]. For smoking exposures, this is doubly concerning because nRBCs have been reported to be increased in the cord blood of newborns with prenatal smoke exposure [13] and also in those born prematurely [14]. Indeed, we and others have had manuscript reviewers suggest, even insist, that methylation changes associated with smoking were indirectly caused by the presence of nRBCs in the cord blood from smoking mothers. However, a failure to find any evidence to support this in the literature has led us to further explore this question.

Methylation levels of several CpGs in the aryl hydrocarbon receptor repressor (*AHRR*) gene have been validated as biomarkers of smoking exposure in adults [15] and neonates [2]. In studies of maternal smoking and methylation as well as adult smoking and methylation, the *AHRR* CpG cg05575921 located at chromosome 5:373378 has consistently been the most significantly associated with smoking and in most studies has the greatest effect size (i.e., difference between nonsmoker and smoker) [5, 16]. *AHRR* plays a key role in the aryl hydrocarbon receptor (AHR) signaling pathway, acting as a negative regulator of AHR. Activation of AHR both mediates the detoxification of the polycyclic aromatic hydrocarbon (PAH) components of tobacco smoke and also regulates various stages in hematopoiesis [17–19], notably, enforcing hematopoietic stem and progenitor cell quiescence [20]. Thus, smoking-associated DNA methylation alterations in hematopoietic cells may not only be biomarkers of exposure but could alter cellular phenotypes and be intermediate in the development of smoking-induced diseases, as has been suggested [5, 7, 21].

To assess if *AHRR* methylation status in nRBCs might influence the measurement of methylation in whole cord blood, we compared *AHRR* DNA methylation levels in whole cord blood with those observed in cord blood-derived CD235a+ nRBCs and CD14+ monocytes and assessed the association with prenatal smoking exposure. We compared methylation levels in whole cord blood with cord blood serum levels of cotinine, the metabolic breakdown product of nicotine. We enumerated nRBC levels in the cord blood samples using both conventional complete blood count (CBC) methods and methylation array-based deconvolution models [11, 22, 23] and then tested if nRBC levels were associated with methylation measurements. We observed highly significant smoking-associated effects on methylation in whole cord blood and in both isolated CD14+ monocytes and CD235a+ nRBCs. However, overall nRBC methylation levels in *AHRR* had no significant impact on *AHRR* methylation measurements in whole cord blood, or on smoking-associated effects, and *AHRR* methylation levels were not associated with nRBC counts.

## Methods

### Population

The cohort of 53 individuals was recruited at WakeMed Health and Hospitals in Raleigh, NC. Pregnant mothers were identified and recruited to the study prior to delivery. A similar proportion of smoking and nonsmoking mothers were matched on maternal age (± 5 years), race, and gestational age (± 2 weeks). In response to a nurse-administered questionnaire, mothers provided a smoking history including self-reported cigarettes per day, years of smoking, and number of cigarettes in the past 24 h and exposure to environmental tobacco smoke, in addition to a standard medical history. Self-reported former smokers were excluded from the study. Demographic information for the full population is provided in Table 1 and Additional file 1: Table S1 has additional information about all subjects and data used for analysis. Demographics for the subset of individuals with 850K data are shown in Additional file 1: Table S2.

**Table 1:** Demographic characteristics of the study participants

	Maternal smoking status		
	Nonsmoker (*n* = 19)	Smoker (*n* = 34)	
	Mean ± SD	Mean ± SD	*p* value (Welch’s *t* test)
Maternal age (years)	27.1 ± 4.9	27.4 ± 4.4	0.82
Gestational age (days)	275.6 ± 7.6	272.0 ± 10.6	0.16
Birthweight (g)	3382.1 ± 262.4	3130.6 ± 423.1	0.01
Cotinine (ng/mL)	2.0 ± 0.0	86.8 ± 85.1	2.43E−06
Cigarettes per day	–	7.9 ± 5.1	–
Years smoked	–	10.9 ± 6.6	–
	Percentage	Percentage	*p* value (two-tail Fisher’s exact test)
Infant sex female	52.6	55.9	0.99
African-American	52.6	53.1	0.99
C section	47.4	64.7	0.38
Progesterone use during pregnancy	5.3	2.9	0.99
Any environmental tobacco smoke (ETS) exposure	10.5	85.3	5.46E−08

### Sample collection and processing

Umbilical cord blood was collected by venipuncture into bags containing the anticoagulant citrate phosphate dextrose. Time between the collection of cord blood and laboratory processing ranged from 3 to 24 h. Cord blood was also collected into Paxgene Blood RNA tubes and serum separation tubes (BD Biosciences). Cotinine/nicotine levels were analyzed in cord blood serum (Quest Diagnostics) for 52 participants; one serum separation tube was lost during processing. Cord bloods with no detectable cotinine were assigned a value of 2 ng/mL which represents the assay limit of detection. Remaining whole cord blood was used for cell type isolation. Mononuclear cells were separated by Ficoll (GE Healthcare) centrifugation from the remaining cord blood. CD235a+ nRBCs and CD14+ monocytes were positively isolated using Miltenyi MicroBeads and Invitrogen Dynabeads, respectively.

### DNA isolations

For separated cell types, DNA was extracted using an AllPrep DNA/RNA/miRNA Universal Kit (QIAGEN). Whole blood DNA was isolated by combining the PAXgene Blood miRNA kit with the DNeasy Blood & Tissue Kit (QIAGEN). Briefly, samples were processed using the PAXgene Blood miRNA kit to the isopropanol precipitation step at which half the sample (~ 700 μl) was loaded onto a DNeasy Mini Spin column and processed using the manufacturer’s instructions for the DNeasy Blood & Tissue Kit followed by concentration with a Microcon-30 kDa Centrifugal Filter Unit (Millipore Sigma). DNA was quantified by a Qubit Fluorometer (ThermoFisher) and stored at − 20 °C.

### Methylation analysis

Bisulfited-converted whole cord blood DNA (*n* = 43) treated with the EZ-96 DNA Methylation MagPrep kit (Zymo Research) was analyzed on the Illumina MethylationEPIC arrays (also known as 850K arrays) following manufacturer’s protocols. Only 43 whole cord blood DNA samples were available at the time of 850K array processing. Two batches were run, one batch (*n* = 41) had 16 nonsmokers and 25 smokers and a second batch (*n* = 2) with one 29-year-old black nonsmoker mother (W068.1) and one 32-year-old black smoking mother (W067.1). Whole cord blood DNA, CD14+, and CD235a+ DNA was bisulfite converted using EZ DNA Methylation-Gold Kit (Zymo Research) following manufacturer’s instructions. Pyrosequencing was used to measure DNA methylation (*n* = 53) at a previously identified [7] smoking-associated differentially methylated region (SM-DMR) containing four CpG sites in the *AHRR* gene located at (chr:5:373378, chr5:373398, chr5:373476, and chr5:373490). The CpG located at chr5:373378 is the same CpG site present on both the Illumina 450k and 850K arrays (cg05575921) which has been widely accepted as a biomarker of smoking in neonatal [2] and adult blood [15, 24]. Two pyrosequencing assays were developed for these four sites using the PyroMark Assay Design Software v2.0 (QIAGEN). DNA methylation reference samples were used to validate each pyrosequencing assay.

All pyrosequencing PCRs contained 20 ng bisulfite-treated genomic DNA, 0.1 μM each primer, 3.5 mM MgCl_2_, 0.4 mM dNTPs, 2.5× DMSO, 1× PCR buffer, and 0.05 units/μL Platinum® *Taq* polymerase (Invitrogen). Thermocycling conditions were 95 °C for 10 min, followed by 5 cycles each of 95 °C/10 s, 64–58 °C/10 s (in 1 °C decreasing increments), 74 °C/15 s, with an additional 15 cycles of 95 °C/10 s, 57 °C/10 s, and 74 °C/15 s. PCR products (15 μL) were sequenced with 0.4 μL of 10 μM sequencing primer using a PyroMark Q96 MD (QIAGEN). Samples were run in triplicate in three separate PCRs performed on different days. Samples were run with 100% and 0% methylation controls (Zymo Research). Average CpG methylation across the SM-DMR was determined from samples with methylation values at each CpG in the SM-DMR.

The *AHRR* pyrosequencing primers are the following: PCR primer F1 TGGGGATTGTTTATTTTTGAGAGG, PCR primer R1 CAACCTATCCCCTACCTCCC, sequencing primer S1-GTTTTGGTTTTGTTTTGTATT (positions chr5:373378, chr5:373398), and sequencing primer S2 GTTTTGGTTTTGTTTTGTATT (positions chr5:373476, chr5:373490). When referring to pyrosequencing results, we use the CpG chromosome position (i.e., chr5:373378), and when referring to 850K results, we use CpG probe ID (i.e., cg05575921).

### 850K data processing and cell type modeling

The raw 850K methylation image files were processed using the minfi package in R [25]. The normal-exponential out-of-band (noob) correction method was used for background correction and dye-bias equalization. Batch correction was carried out using champ.runCombat function in the ChAMP package. Probes containing polymorphic CpGs were filtered out using champ.filter function in the ChAMP package [26]. The methylation level at each CpG was reported as the beta value [*β* = intensity of the methylated allele (M)/(intensity of the unmethylated allele (*U*) + intensity of the methylated allele (*M*) + 100)]. For purposes of *AHRR* methylation in this project, only normalized, batch-corrected methylation values at CpG chr5:373378 (cg05575921 on the 850K array) are reported. Normalized and batch-corrected methylation values for cell type-specific CpGs were used to estimate cell type percentages (CD4+ T cells, CD8+ T cells, B cells, monocytes, granulocytes, natural killer cells, and nRBCs) using the method of Houseman et al. [22] as modified [11, 23]. The modification adds a group of nRBC specific CpGs to the model allowing the calculation of an estimated percentage of nRBCs in the whole cord blood sample.

### Statistical analysis

To minimize multiple comparisons in our assessment of smoking-associated methylation in our cell type samples, the methylation analyses included only methylation percentages of four CpG sites from pyrosequencing and one CpG, cg05575921 from 850K arrays. Differences between methylation levels of smokers and nonsmokers at the *AHRR* CpGs were first assessed by a two-sided Welch’s *t* test. We compared results from pyrosequencing and 850K arrays (reported as *r*^2^) using univariable linear regression in GraphPad Prism (GraphPad Software). The association between methylation and cotinine was also assessed using univariable linear regression in GraphPad Prism. All data was normally distributed within groups allowing parametric statistics. For multivariable models, given the relatively small sample size and potential impact of outlier values, we tested associations with cg05575921 methylation using robust linear regression (using M estimation) (PROC ROBUSTREG, SASv9.4). Three multivariable models testing methylation versus smoking status were adjusted for gestational age, infant sex, and race, as well as adjusted for (model 1) six cell types’ percentages (CD8+ T cells, CD4+ T cells, natural killer cells, B cells, granulocytes, and monocytes) based on the Houseman model [22] or (model 3) as modified [11, 23]. We also ran the six cell type model adjusted for CD235a+ cell counts (model 2, log10nRBC_abs_) using the adjusted nRBC number (1.0E + 03 cells/μL) from a complete blood count with a log10 transformation. Zero nRBC values were imputed by log10 transforming the limit of detection (i.e., lowest nRBC value) divided by the square root of two. Robust linear regression models were also run following the transformation of methylation beta values into *M* values (*M* = log_2_ (beta/1-beta)). Statistical significance levels for each analysis following multiple comparison adjustment are noted in each table.

### Replication studies

We searched Gene Expression Omnibus (GEO) Datasets and used two public datasets for replication our finding. First, we used GSE127824 GEO [27], which generated DNA methylation profiles using Illumina 450k array for 24 whole cord blood samples from healthy children born via caesarian section with matched fluorescence-activated cell sorting (FACS) counts of nRBCs. We extracted cg05575921 methylation beta values and tested the association between measured nRBC counts in these samples and *AHRR* cg05575921 methylation levels. Second, we fetched GSE88929 which has Illumina 450k DNA methylation profiles from a larger sample of full-term cord bloods (*n* = 114) [28] and modified the minfi estimateCellCounts function to take nRBC methylation beta values as input and the “FlowSorted.CordBlood.450k” as reference [11, 23] to deconvolute and estimated nRBC and other cell content in these samples. We then tested if deconvolution estimated nRBC percentage was associated with *AHRR* cg05575921 methylation using univariable linear regression.

## Results

### Prenatal tobacco smoke exposure and DNA methylation

Fifty-three participants had complete pyrosequencing methylation data for 4 CpGs across an *AHRR* smoking-associated differentially methylated region (SM-DMR) measured in DNA extracted from whole cord blood, CD14+ monocytes, and CD235a+ nRBCs (Table 1, Additional file 1: Table S1). Of these individuals, 52 had cotinine analyses measured in cord blood serum as a biomarker of very recent smoking. No self-reported nonsmokers had detectable cotinine in their cord blood while 26 of 33 smoking mothers had cotinine levels > 2 ng/mL (detection limit), indicating some level of recent tobacco smoke exposure. Seven of the cord bloods from self-reported smoking mothers had very low cotinine (≤ 8 ng/mL), suggesting they had not smoked recently.

**Fig. 1:**
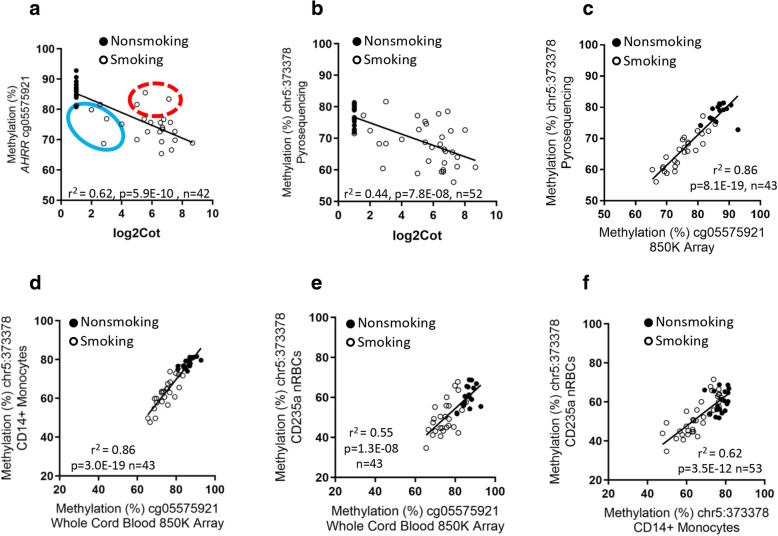
Log2 cotinine measurement in cord blood serum versus DNA methylation at cg05575921 (chr5:373378) measured by **a** 850K array and **b** pyrosequencing in cord blood DNA. Red circle indicates high cotinine relative to the methylation effect and blue circle indicates low cotinine relative to the methylation level. **c** Comparison of DNA methylation at cg05575921 measured by 850K array (*x*-axis) and pyrosequencing (*y*-axis) in cord blood DNA. **d**–**f** Comparison of DNA methylation at cg05575921 measured by 850K array in cord blood with methylation at chr5:373378 (genomic position of cg05575921) measured by pyrosequencing in DNA from: **d** CD14+ monocytes, and **e** CD235a+ nRBCs. **f** Comparison of methylation at chr5:373378 in CD14+ monocytes with CD235a+ nRBCs

Among the participants with cord blood DNA evaluated by 850K array, the CpG at chr5:373378 (cg05575921) was significantly correlated with cotinine measured in cord blood serum (*r*^2^ = 0.62, *p* = 5.9E−10, *n* = 42) (Fig. 1a) and was associated with self-report of smoking (yes/no) during pregnancy (*t* test, *p* = 2.1E−11). We observed a similar relationship in the 52 subjects with cotinine and methylation measurements at chr5:373378 by pyrosequencing (Fig. 1b). There was considerable variation in the cotinine levels in the cord blood from smokers relative to levels of methylation at cg05575921 (Fig. 1a, b). There is an established relationship between maternal smoking in the third trimester of pregnancy [4, 5] and *AHRR* methylation, and we observed several participants who had high cotinine relative to the methylation effect (red circle) and some that had low cotinine relative to the methylation level (blue circle). Methylation at *AHRR* chr5:373378 was significantly correlated with self-reported cigarettes per day (univariate linear regression, *r*^2^ = 0.27, *p* = 6.62E−05, *n* = 52; Additional file 2: Figure S1), but this was less significant than the correlation with cord blood serum cotinine concentration or smoking (yes/no). Absolute nRBC counts were not associated with cotinine (*r*^2^ = 0.005, *p* = 0.59) or gestation age (*r*^2^ = 0.002, *p* = 0.75).

Among the 43 subjects with methylation data in cord blood DNA from both 850K cg05575921 (Additional file 1: Table S2) and pyrosequencing methods (chr5:373378), we observed a highly significant correlation between measurements by these two techniques (Fig. 1c, *r*^2^ = 0.86; *p* = 8.1E−19). This direct comparison showed that methylation values determined by pyrosequencing at the same CpG, chr5:373378, while highly correlated to array values, averaged about 8% lower (8.8% mean difference, *p* = 1.9E−07). We assessed how methylation levels at cg05575921 in cord blood by array compared with methylation measured by pyrosequencing in both CD14+ monocytes (Fig. 1d, *r*^2^ = 0.86, *p* = 3.0E−19) and CD235a+ nRBCs (Fig. 1e; *r*^2^ = 0.55, *p* = 1.3E−08). These comparisons indicated greater variability in nRBC methylation levels than in CD14+ monocytes, relative to whole cord blood.

Figure 1f displays the correlation of *AHRR* methylation at the chr5:373378 CpG in CD14+ monocytes with methylation levels in CD235a+ nRBCs among the 53 subjects with pyrosequencing data (Fig. 1f. *r*^2^ = 0.62, *p* = 3.5E−12). *AHRR* methylation levels in the two cell types were significantly correlated but lower in CD235+nRBCs. Specifically, methylation values at chr5:373378 in each subject averaged 14.6% lower in CD235a+ nRBCs (range 0.4 to 24.8%, *p* = 3.8E−13) relative to CD14+ monocytes. Stratifying individuals by prenatal smoke exposure we observed that the cell-type specific difference in methylation at chr5:373378 between CD14+ monocytes and CD235a+ nRBCs was significantly greater in nonsmokers (− 17.2%) than it was in smokers (− 13.1%, *p* = 1.8E−02).

**Fig. 2:**
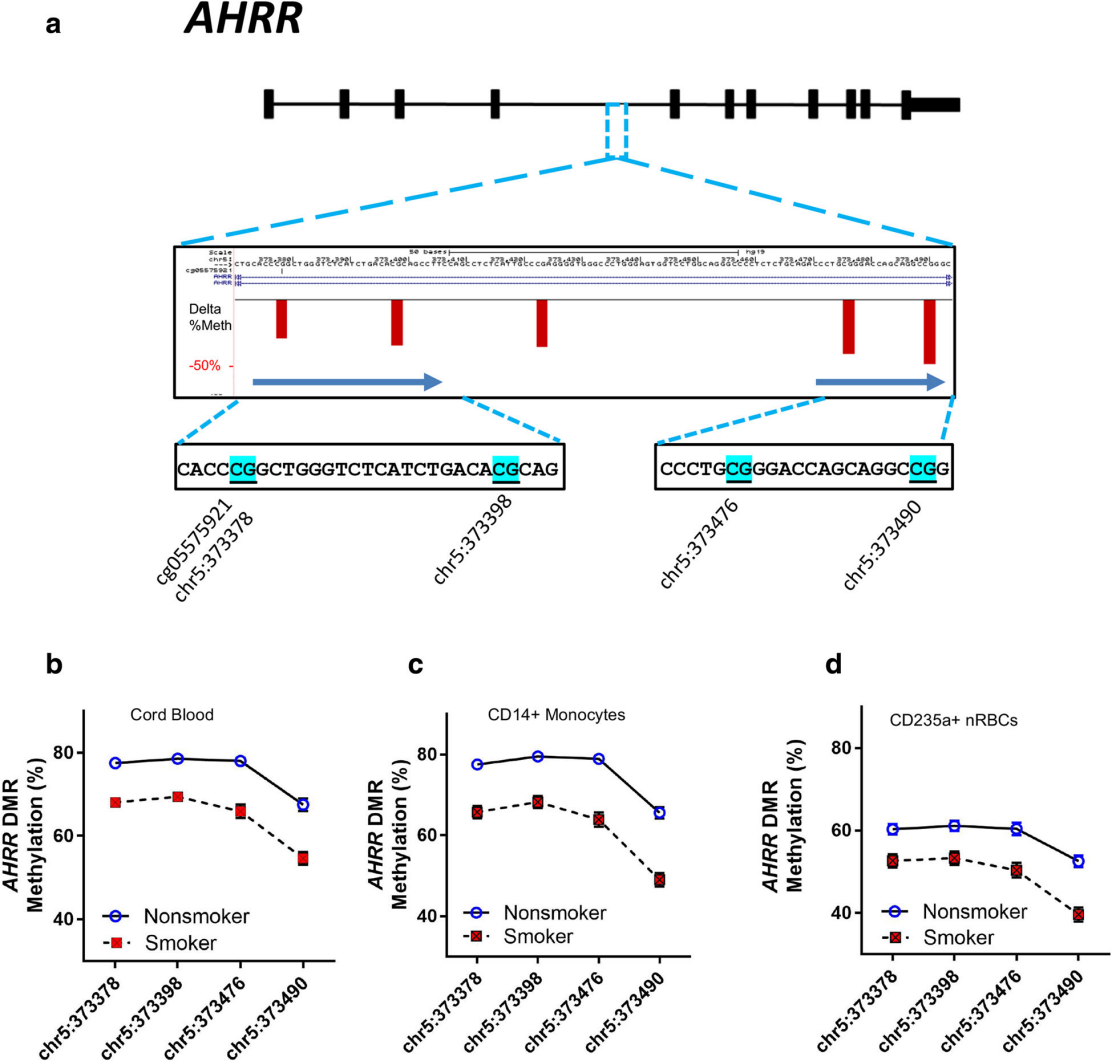
**a** Browser view of *AHRR* SM-DMR with genomic sequence for 4 CpGs measured by pyrosequencing that displays smoking-associated hypomethylation. The red vertical bars indicate percent hypomethylation at CpGs in this *AHRR* DMR region that are associated with smoking in adults [7]. The CpG at chr5:373378 is referred to as cg05575921 on Illumina 450K and 850K arrays. **b**–**d** Pyrosequencing measurements of 4 *AHRR* CpGs (mean, +/− SEM) for prenatal smoke exposure and nonsmokers measured in DNA from **b** whole cord blood, **c** CD14+ monocytes, and **d** CD235a+ nRBCs

Figure 2a displays the genomic location of the *AHRR* CpGs measured by pyrosequencing. Pyrosequencing-based methylation levels from samples grouped into nonsmoking and smoking mothers at each *AHRR* CpG site in the SM-DMR for each cell type are shown in Fig. 2b–d. At these 4 CpGs, we observed a decrease in methylation (range − 7.7 to − 16.7%) associated with prenatal smoke exposure measured in whole cord blood, CD14+ monocytes, and CD235a+ nRBCs (Table 2), with the largest and most significant decrease occurring in CD14+ monocytes at the CpG located at chr5:373490 (− 16.7%, *p* = 7.6E−10), 112 nt downstream of chr5:373378 (cg05575921) (Table 2).

**Table 2:** Pyrosequencing analysis of individual CpGs in whole cord blood, CD14+ monocytes, and CD235+ nRBCs

Chromosome position	Whole cord blood				CD14+ monocytes				CD235a+ nRBCs			
	chr5:373378	chr5:373398	chr5:373476	chr5:373490	chr5:373378	chr5:373398	chr5:373476	chr5:373490	chr5:373378	chr5:373398	chr5:373476	chr5:373490
Methylation (%), nonsmoker (*n* = 19), mean ± SEM	77.5 ± 1.3	78.6 ± 1.4	78.0 ± 1.8	67.5 ± 1.8	77.5 ± 1.6	79.5 ± 1.7	78.9 ± 2.0	65.6 ± 2.0	60.3 ± 1.9	61.1 ± 1.9	60.4 ± 2.1	52.5 ± 2.0
Methylation (%), smoker (*n* = 34), mean ± SEM	68.1 ± 1.0	69.4 ± 1.0	65.9 ± 1.4	54.6 ± 1.4	65.7 ± 1.2	68.2 ± 1.3	63.9 ± 1.5	49.0 ± 1.5	52.6 ± 1.4	53.3 ± 1.4	50.4 ± 1.6	39.6 ± 1.5
Delta methylation (%)	− 9.4	− 9.2	− 12.2	− 12.9	− 11.8	− 11.3	− 15.0	− 16.7	− 7.7	− 7.8	− 10.0	− 12.9
*p* value (two-sided *t* test)	7.0E−09*	7.8E−08	5.5E−08	1.8E−07	5.7E−09	7.8E−08	2.2E−09	7.6E−10	4.8E−04	4.8E,04	8.5E−05	3.2E−07

*The statistical significance cutoff following adjustment for multiple comparisons is *p* < 0.0125

### Modeling smoking effects and cell type composition

To test the relationship between absolute nRBC counts or estimated nRBC percentage impacted *AHRR* methylation levels in whole cord blood, we tested three models using robust multivariable regression analysis and assessed the models by examining the smoking parameter estimate *p* value and the adjusted *r*^2^ (*r*^2^_adj_) (Table 3). Full robust model result output is provided in Additional file 1: Tables S3–S5. The default model 1, used in many methylation studies of smoking in cord blood, tested smoking (yes/no) versus methylation at cg05575921 and adjusts for demographic variables (gestational age, race, sex of the infant), and estimated cell-type percentages for six leukocyte cell types (CD4+ T cells, CD8+ T cells, B cells, monocytes, granulocytes, and natural killer cells) [22]. This model had a highly significant *p* value for the smoking parameter estimate (*p* = 2.11E−20) and explained 60% of the variance (*r*^2^_adj_ = 0.60).

**Table 3:** Robust multivariable regression analysis of cg05575921 methylation association with smoking

Model	Model co-variates	Smoking parameter estimate	Smoking parameter estimate, *p* value*	Model adjusted *r*^2^
Model 1. Adjusted for 6 cell types (estimated without nRBC_est_)	cg05575921 = smoking status + gestational age + infant sex + race + CD8 T cells + CD4 T cells+ natural killer cells + B cells + monocytes + granulocytes	− 0.1284	2.11E−20	0.60
Model 2. Adjusted for 6 cell types (estimated without nRBC_est_) and log10nRBC_abs_	cg05575921 = smoking status + gestational age + infant sex + race +CD8 T cells + CD4 T cells+ natural killer cells + B cells + monocytes + granulocytes + log10nRBCs_abs_	− 0.1388	1.69E−49	0.66
Model 3. Adjusted for 7 cell types (including nRBC_est_)	cg05575921 = smoking status + gestational age + infant sex + race + CD8 T cells + CD4 T cells+ natural killer cells + B cells + monocytes + granulocytes + nRBC_est_	− 0.1251	1.69E−33	0.61

Models 1 and 2 used cell type percentages calculated with the 6 cell-type de Goede deconvolution model [11]

*The statistical significance cutoff following adjustment for multiple comparisons is *p* < 0.0125

We tested the effect of adding nRBC counts from CBC analysis (model 2, log10nRBCs_abs_) to the 6-cell type model (model 1), and this combination of adjustments provided the strongest explanatory model (based on significance of smoking parameter estimate, *p* = 1.69E−49 and *r*^2^_adj_ = 0.66). The parameter estimate for log10nRBCs_abs_ was significant and slightly positive suggesting higher levels of nRBCs were associated with higher methylation levels in cord blood.

We then used a set of nRBC-specific CpGs to estimate nRBC percent (nRBC_est_) in whole cord blood in a seven cell-type deconvolution model as in Bakulski et al. [23] and others [11, 29]. Adding adjustment for methylation-based, estimated cell-type composition to model 1 (model 3, 7 cell types) modestly improved the correlation (i.e., improved *r*^2^_adj_ from 0.60 to 0.61, explaining ~ 1% more of the variance) with methylation levels and increased the *p* value of the smoking parameter estimate (Table 3, Additional file 1: Tables S2–S5).

Given that we had nRBC counts from CBC analysis (absolute nRBC × 1.0E+ 03 cells/μL; nRBC_abs_), we tested how well nRBC_abs_ counts correlated with the deconvolution estimated nRBCs (nRBC_est_ versus log10nRBC_abs_ (*r*^2^ = 0.27, *p* = 4.3E−04; Additional file 2: Figure S2). While the association was highly significant, the relatively low *r*^2^ suggests that either the nRBC measurement or the estimation method has considerable variability.

### Replication study

Testing the association between measured absolute nRBC levels in 24 cord blood samples and *AHRR* cg05575921 methylation in GEO GSE127824 [27], we observed no association (*p* = 0.34; Additional file 2: Figure S3a). In a second independent dataset (GEO GSE88929) [28], we tested if deconvolution-estimated [11, 23] percent RBCs differed among nonsmokers and smokers and they did not (*p* = 0.87; Additional file 1: Table S6). We also test if estimated nRBCs were associated with *AHRR* cg05575921 methylation using univariable linear regression and we observed no significant association (*p* = 0.46; Additional file 2: Figure S3b).

## Discussion

Our small study confirms that self-reported maternal smoking is strongly associated with changes in *AHRR* DNA methylation in whole neonatal cord blood DNA [4, 5], and to our knowledge, this is the first report in which highly significant smoking effects are observed in cord blood-derived CD14+ monocytes and CD235a+ nRBCs. This is also the first report in which cotinine levels measured in serum derived from cord blood are associated with loss of methylation in the *AHRR* SM-DMR. We observed that some cord blood samples had relatively high levels of cotinine relative to the expected *AHRR* methylation level while other samples displayed smoking-associated methylation differences but little or no measured cotinine. Cotinine is known to be a short-term biomarker of tobacco exposure, so individuals with high cotinine may have very recently used tobacco or an electronic nicotine delivery device (e.g., e-Cigarette). Conversely, those with low cotinine relative to the *AHRR* methylation level may have reduced their smoking as their expected delivery date neared. No self-reported nonsmokers had cotinine in their serum or had cg05575921 levels suggesting that they were smokers or former smokers. However, based on the reports from Joubert et al. [3] and Reese et al. [2], if former smokers were present in the nonsmoking group, it would be difficult to detect smoking effects at cg05575921 in cord blood DNA.

De Goede et al. [11] demonstrated that nRBCs from cord blood are hypomethylated relative to whole cord blood DNA and that they heterotopically interact with other hematopoietic cell types, particularly T cells [8]. The same authors raise the concern that nRBC’s presence in whole blood can impact methylation levels [8]. It is known that maternal smoking is associated with increased numbers of nRBCs in cord blood [13] and increased nRBC counts in cord blood have been suggested as a marker of hypoxia [30]. Thus, we considered the hypothesis that higher levels of nRBCs in the cord blood of prenatally smoke-exposed neonates might confound the measurements of smoking-associated differential methylation in whole cord blood. We examined the correlation of smoking-associated CpGs in whole cord blood and isolated CD235a+ nRBCs, also comparing these with CD14+ monocytes, a cell type well known to display SM-DMRs [6, 7, 21]. Methylation levels of *AHRR* smoking-associated CpGs were significantly lower in CD235a+ nRBCs relative to whole blood and CD14+ cells, with some individuals showing much larger differences (mean difference − 14.6%, range 0.4 to 24.8%) than others. Lower methylation values for *AHRR* in nRBCs relative to CD14+ monocytes were observed in both smoking-exposed and unexposed neonates, and surprisingly, the cell type difference in methylation was significantly greater in unexposed neonates, suggesting that the lower methylation levels in nRBCs are independent of smoking exposure. We hypothesize that the lower methylation levels of *AHRR* in nRBCs relative to monocytes and whole cord blood is due the presence, in each isolated CD235a+ nRBC sample, of a small percentage of nRBCs that have fully demethylated their genomes.

While our model results suggested a modest positive effect of nRBC counts on *AHRR* methylation levels, the replication analysis in two other independent datasets was not consistent with this observation. This leads us to conclude that it is useful to include nRBC counts or estimates in models of smoking effects on cord blood methylation, but it is unlikely that nRBCs directly impact smoking-associated methylation alterations in full-term cord blood. Perhaps, there are other causes of elevated nRBCs that are independent of maternal smoking.

Methylation of the CpG cg05575921 is known to be strongly associated with tobacco smoke exposure in both adults and neonates. Our previous study using reduced representation bisulfite sequencing (RRBS) analysis of *AHRR* in adults revealed that several nearby CpGs in *AHRR* that are not on the 450 or 850K arrays show similar or greater smoking associated effects [7]. For example, in adult CD14+ monocytes, methylation at cg05575921 (chr5:373378) showed a loss of − 37.7% while the CpG at chr5:373490 (112 nt downstream) showed a greater loss of 54.4% (*p* = 9.0E−13) [7]. The present data in cord blood CD14+ monocytes is consistent with this difference between CpG loci observed in adults. For example, the CpG at chr5:373378 showed a loss of − 11.8% (*t* test, *p* = 5.7E−09), while the CpG at chr5:373490 showed a greater loss of − 16.7% (*p* = 7.6E−10) (Table 2).

Smoking-associated loss of methylation in *AHRR* in cord blood CD14+ monocytes was somewhat less than our previous findings in adult blood monocytes (e.g., at cg05575921 a loss of − 11.8% in cord blood versus − 37.7% in adults), but this difference was consistent with cg05575921 results in studies of whole cord blood by array. This effect size difference may be due to lower levels of tobacco use in pregnant women relative to adult smokers, particularly in the last trimester, and this would be consistent with cord blood *AHRR* methylation levels observed in some of our self-reported smokers that were very similar to nonsmokers. Also, the effective dose of the causal factor in tobacco smoke resulting in the methylation effect is likely to be lower in the fetal circulation relative to adult smokers, who get their exposure directly into the pulmonary circulation.

The size of this study is a limitation; however, we observed highly significant prenatal tobacco smoke-associated changes in *AHRR* methylation levels in whole cord blood, CD14+ monocytes, and also in CD235a+ nRBCs. Any of these cell types could potentially be used as a source of DNA to detect smoking-associated alteration of *AHRR* methylation. However, depending on the quantity of nRBCs present in the newborn blood and their stage in erythrocyte development and enucleation, the quantitation of methylation in the CD235a+ nRBC fraction might be strongly affected. Our robust regression models with the inclusion of estimated cell-type composition, including nRBC counts or estimates, showed marginally increased correlation (*r*^2^) and greater significance with regard to the prenatal smoke-exposure effect on *AHRR* cg05575921 methylation. While it was not possible to analyze the two replication datasets exactly as done in our experiment, in the replication neither absolute nRBC counts or estimated nRBCs percentage were significantly correlated with methylation levels at *AHRR* cg05575921, suggesting nRBCs do not modulate the effects of smoking on DNA methylation. However, in studies of preterm neonates, who display very high percentages of nRBCs, the effects of nRBC quantity on DNA methylation measurements in whole cord blood could be very important. This study confirms that prenatal tobacco smoke exposure strongly affects DNA methylation in the *AHRR* gene and is the first study to demonstrate that a prenatal smoke exposure biomarker is largely unaffected by cord blood cell type composition in full-term neonates.

## Conclusions

Prenatal smoke exposure was highly significantly associated with *AHRR* methylation in cord blood, CD14+ monocytes, and CD235a+ nRBCs. *AHRR* methylation levels in nRBCs and nRBC counts had minimal effect on methylation measurements in whole cord blood. However, regression models using estimated nRBCs or actual nRBC counts modestly outperformed those lacking these covariates.

This study was approved by the Institutional Review Board of WakeMed UNC Hospitals and the NIH. All subjects provided written consent to participate and were recruited under IRB approved protocol, WakeMed #678553-2.

## Additional files

Additional file 1: Supplemental Tables S1-S6. (XLSX 59 kb)

Additional file 2: **Figure S1.** *AHRR* methylation at chr5:373378 by pyrosequencing in cord blood versus self-reported maternal cigarettes per day. **Figure S2.** Log10 absolute nRBC count vs deconvolution estimated nRBC percentage. **Figure S3.** a Absolute nRBCcount from cord blood vs *AHRR* cg05575921 methylation (%) Jones MJ et al (GSE127824), b Deconvolution estimated nRBC percentage from cord blood GSE88929 vs . *AHRR* cg05575921 methylation (%) Haertleet al (GSE88929). (PDF 633 kb)

## Data Availability

The dataset(s) supporting the conclusions of this article are included within the article and its additional files. See Additional file 1: Table S1 for methylation values for cg05575921 and pyrosequencing CpGs for each participant.
