# Supplementary material for: Smoking-associated AHRR demethylation in cord blood DNA: impact of CD235a+ nucleated red blood cells
Source: Clin Epigenetics. 2019 Jun 10;11:87. doi: 10.1186/s13148-019-0686-1 (PMC6558773; doi:10.1186/s13148-019-0686-1)
Supplement: Supplementary file 2 — Figure S1. AHRR methylation at chr5:373378 by pyrosequencing in cord blood versus self-reported maternal cigarettes per day. Figure S2. Log10 absolute nRBC count vs deconvolution estimated nRBC percentage. Figure S3. a Absolute nRBCcount from cord blood vs AHRR cg05575921 methylation (%) Jones MJ et al (GSE127824), b Deconvolution estimated nRBC percentage from cord blood GSE88929 vs . AHRR cg05575921 methylation (%) Haertleet al (GSE88929). (PDF 633 kb) [file 13148_2019_686_MOESM2_ESM.pdf]

Additional File 2

Figure S1

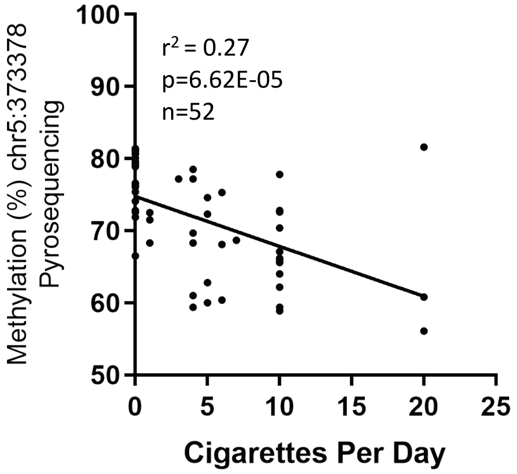

Additional File 2 Fig. S1 AHRR methylation at chr5:373378 by pyrosequencing in cord blood versus self-reported maternal cigarettes per day.

Figure S2

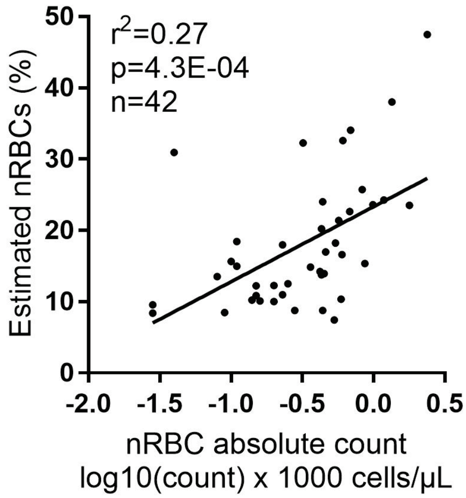

Additional File 2 Fig. S2 Log10 absolute nRBC count vs deconvolution estimated nRBC percentage.

Figure S3

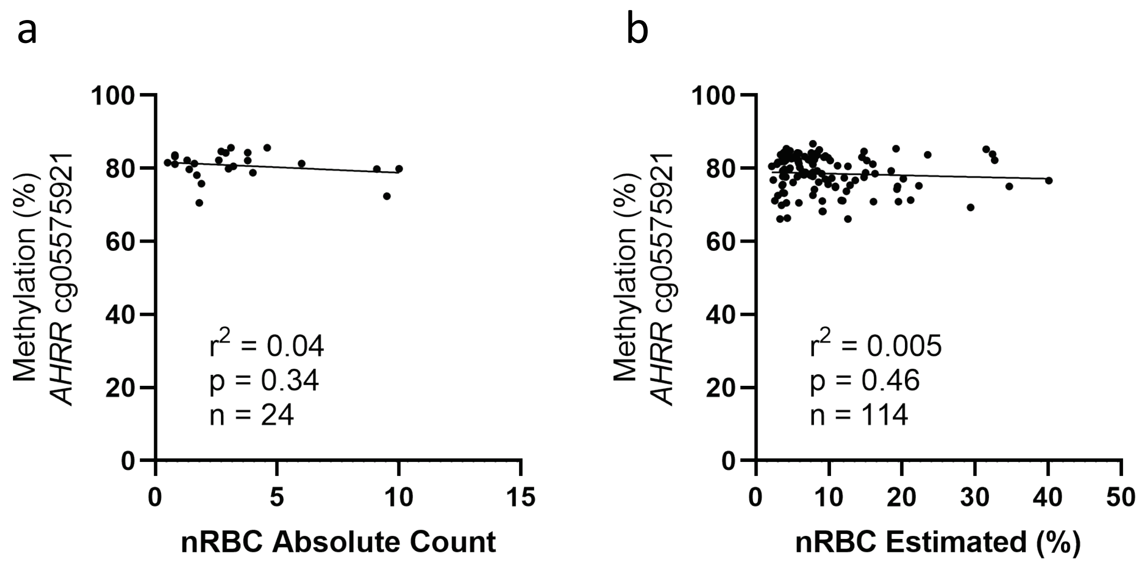

Additional File 2 Fig. S3 a Absolute nRBC count from cord blood vs *AHRR* cg05575921 methylation (%) Jones MJ et al (GSE127824), b Deconvolution estimated nRBC percentage from cord blood GSE88929 vs . *AHRR* cg05575921 methylation (%) Haertle et al (GSE88929).
